# Comparative evaluation of long-term ophthalmic sequelae following first versus second-line antibiotic treatment for congenital syphilis

**DOI:** 10.1186/s40942-025-00689-y

**Published:** 2025-06-10

**Authors:** Samuel Montenegro Pereira, Maria Alix Leite Araújo, Rian Vilar Lima, Rivianny Arrais Nobre, Ana Patrícia Alves da Silva, Ana Fátima Braga Rocha, Rodrigo Jorge, Nathalie Broutet

**Affiliations:** 1 Center for Health Sciences, University of Fortaleza, Ceará, Brazil; 2 Maternal and Child Health Department, Federal Ceara University, Ceará, Brazil; 3 Ophthalmology Division, Ribeirão Preto Medical School, University of São Paulo, Ribeirão Preto, Brazil; 4 Consultant in Health Sciences Research, Geneva, Switzerland

**Keywords:** Pediatric retina, Congenital syphilis, Optic atrophy

## Abstract

**Purpose:**

Congenital syphilis (CS) is associated with interstitial keratitis, chorioretinitis, uveitis, and optic atrophy mainly in inadequately treated patients. We conducted a retrospective cohort analysis evaluating ocular findings in children born in 2015 with CS treated with ceftriaxone at the time of delivery during the period of penicillin shortage in a city located at Northeast of Brazil and compared them with those adequately treated.

**Methods:**

469 children were reported with CS at birth during the penicillin shortage period and 171 were actively searched and invited to an ophthalmological assessment and retrospective analysis of their information recorded in the medical records of the municipality’s health services.

**Results:**

A total of 68 children came to the assessment, median age 8 years of age (range 7–8 years), 48 were treated with penicillin (70.5%) and 20 with ceftriaxone (29.5%). There were no significant differences in demographic or perinatal characteristics between the groups. The majority of children had a completely normal ophthalmological examination (67.6%). Regarding findings that are more associated with CS, one child in ceftriaxone group (5.0%) had optic atrophy in one eye and one in the penicillin group (2.9%) had glaucomatous optic disc changes. No interstitial keratitis was found. There was no significant association between the child’s treatment and the prevalence of ophthalmologic findings (*p* = 0.663). There was also no association between the medication and a current reactive VDRL (*p* = 1.000).

**Conclusion:**

After an 8-year follow-up, no statistically significant difference was observed in the incidence of ophthalmologic manifestations among individuals treated for CS with either penicillin or ceftriaxone. These findings suggest that ceftriaxone may serve as an effective alternative for the prevention of CS and its associated ocular complications.

## Background

Congenital syphilis (CS) is a major global public health problem with more than 700,000 cases registered in 2022 [1]. The World Health Organization (WHO) estimates that syphilis prevalence among pregnant women has nearly doubled between 2016 and 2022, as a result of this, rates of CS are still on the rise [2].

Despite being associated with interstitial keratitis, chorioretinitis, uveitis, congenital cataract, and optic atrophy the exact incidence of ophthalmic complications associated with CS is uncertain [3]. Among inadequately treated patients, the incidence of ophthalmic manifestations increases until puberty [4]. Epidemiological studies in children are scarce, but works in adult patients with any type or stage of syphilis have found ocular manifestations in 0.53–2.6% of cases, but there is reasonable consensus that this figure is underestimated [3].

Since penicillin was first used to treat syphilis in 1943, it has been the standard treatment for syphilis, and for prevention of CS [5] until today. However, between 2014 and 2016, Brazil and 38 other countries experienced penicillin shortages, forcing them to use second-line therapeutic regimens in more than half of the infants diagnosed with CS [6]notably ceftriaxone, with little or no empirical evidence for efficacy.

Given the uncertainty about the efficacy of ceftriaxone in the treatment of CS to prevent late ocular complications of CS, our aim was to actively search for children treated for CS at birth with either ceftriaxone or penicillin in 2015, carry out a comprehensive ophthalmologic evaluation, and compare clinical findings between treatment groups.

## Methods

### Design, study location and period

This is a cross-sectional study of children notified with CS at birth during a period of penicillin shortage. In Brazil, CS is a notifiable condition, mandating compulsory reporting by healthcare providers to public health authorities for epidemiological surveillance. Relevant data are securely stored and managed by municipal and federal health agencies.

The study protocol received ethical clearance from the Institutional Review Board under protocol number 2.110.189, dated October 4, 2023. Additionally, the Fortaleza Municipal Health Department authorized data access through an official letter of consent. Written informed consent was obtained from all legal guardians and participants, in accordance with national ethical guidelines. Following ethical approval, the research team accessed health records from the Fortaleza Municipal Health Department.

Fortaleza, capital of the state of Ceará in Northeast Brazil, is a metropolitan area with an estimated population of 2,687,000 and a reported CS incidence of 13.5 cases per 1,000 live births in 2015 [7]. In the field of health, the municipality is divided into six health districts whose function is to exercise health authority in the territory, plan, execute, follow up, monitor and evaluate health actions and services at regional level in line with municipal rules and guidelines.

Participants were recruited through direct visits at home by the local Community Health Workers (CHW) and to establish contact with parents or legal guardians, aiming to schedule comprehensive clinical assessments, including ophthalmologic evaluation and laboratorial tests (treponemal rapid test, VDRL and HIV testing). When initial contact attempts failed, the CHW made up to four visits to the homes of potential participants to summon them or reschedule the evaluation date in the event of their absence. The researchers covered the cost of travel to the assessment center.

### Population, inclusion and exclusion criteria

All children notified with CS during the year of 2015 who lived in the two regional health districts closest to the assessment center at the period that research took place were included. The rationale underlying this geographical criterion was that previous studies had already shown that these were patients in highly vulnerable socioeconomic conditions [8] and with great difficulties in adhering even to the standard CS follow-up [9]. Notably, the factor most related to not completing follow-up for CS was the fact that it took place in a hospital other than the one where the child was born, which was typically a more distant facility [9].

The analysis considered children who received penicillin benzathine benzylpenicillin 50,000 IU/Kg, intramuscular, single dose; benzylpenicillin potassium/crystalline 50.000 IU/Kg, intravenously, 12/12 h in the first week of life and 8/8 h from 8 days of life, for ten days; benzylpenicillin procaine 50,000 IU/Kg, intramuscularly, once a day for 10 days; or, alternatively, ceftriaxone 50 mg/kg/day, intravenously or intramuscularly, once a day, for 10 days.

Children who received mixed treatment regimens of ceftriaxone and penicillin combined with each other or with any other antibiotic were excluded.

### Ophthalmological examination

A comprehensive ophthalmic evaluation was conducted for all patients from May to August 2023 and included Snellen Chart visual acuity test, anterior segment biomicroscopy and indirect binocular ophthalmoscopy.

At this stage, the attending ophthalmologist also conducted a review of each child’s medical records, including documentation of the red reflex screening, initial ophthalmologic evaluation performed in the maternity ward, and any available follow-up ophthalmologic data.

An alteration in the ophthalmic neonatal screening test refers to any abnormal finding in the red reflex test, routinely performed within 72 h of birth in Brazil, that suggests conditions such as congenital cataract or retinoblastoma. Visual axis alteration refers to any obstruction or misalignment of the visual axis, including opacities in the cornea or lens that impair the transmission of light to the retina. The test is carried out using the light beam of the direct ophthalmoscope and is considered normal when a bilateral and symmetrical red reflex of the retina is observed.

### Data analysis

Descriptive analysis used counts and percentages for qualitative variables and medians for quantitative ones, with normality assessed by the Shapiro-Wilk test. To evaluate differences between penicillin and ceftriaxone groups we performed student’s t-test or Mann-Whitney U-test for continuous variables, while chi-square or Fisher’s exact test evaluated qualitative differences, with significance at α = 0.05. Analysis was performed using SPSS v20.

## Results

**Fig. 1:**
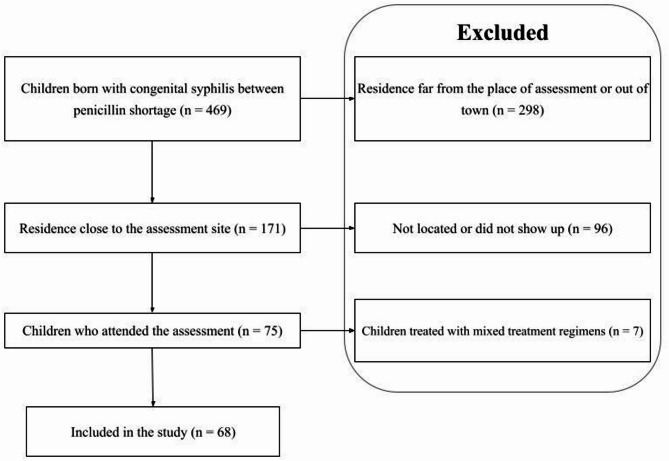
Flowchart of patient inclusion in the study

In 2015, 469 children were reported with CS at birth in Fortaleza, of these, 171 were identified as residing in the two districts closest to the assessment center and invited to be evaluated. A total of 75 children attended the assessments, and for analysis purposes, the patients were divided into two groups: (1) treated with penicillin-based regimens (2) treated with ceftriaxone, at delivery. Seven children received mixed treatments (penicillin + ceftriaxone) and were excluded from the analysis (Fig. 1).

A total of 68 (39.7%) children were treated with the drugs of interest and, therefore, included in the analysis. Mean mother’s age at time of delivery was 24 years (15–44), with 76.4% of them having less than 10 years of formal education and 13.2% reporting drug use. Children were born with a median gestational age of 39 weeks (29–42) and a median birth weight of 3205 g (830–4430). Presented some sign or symptom related to CS at birth 25 children (36.7%), of which 12 (17.6%) had low birth weight, 12 (17.6%) jaundice, 8 (11.7%) prematurity, 2 (2.9%) hepatomegaly, and 1 (1.4%) splenomegaly. There was no association between VDRL titration and the presence of symptoms at birth (*p* = 0.273).

Detailed demographics divided by subgroups of treatment can be found at Table 1.

**Table 1:** Characteristics of mothers and children with CS divided by treatment administered in the maternity ward

Characteristic	Category	Treatment				*p*-value
		Penicillin (*n* = 48)		Ceftriaxone (*n* = 20)		
		Count	%	Count	%	
Mother’s age at delivery		24 (15–44)		22 (16–38)		0.302
Ethnicity	White	3	6.2%	0	0.00%	0.550
	Brown and indigenous	45	93.8%	20	100.0%	
Household income	No income	2	4.2%	1	5.0%	0.789
	≤ 1 minimum wage (230$)	30	62,5%	14	70.0%	
	> 1 minimum wage (230$)	16	33.3%	5	25.0%	
Mother’s treatment	Adequate^a^	16	33.3%	5	25.0%	0.366
	Inadequate or untreated	17	36.2%	10	50.0%	
Preterm		6	12.5%	2	10.0%	1.000
Symptoms at birth^b^		17	36.2%	8	40.0%	0.622
Newborn’s sex	Male	25	52.1%	10	50.0%	0.610
	Female	23	47.9%	10	50.0%	
VDRL at birth	1:1	13	27.0%	4	20.0%	0.452
	1:2	10	20.8%	3	15.0%	
	1:4	4	8.3%	1	5.0%	
	1:8	4	8.3%	3	15.0%	
	1:16	1	2,0%	2	10.0%	
	1:32	1	2,0%	0	0.00%	
	1:64	1	2.0%	0	0.00%	
	1:128	1	2.0%	0	0.00%	
Changes in CSF/Neurossífilis^c^		3	6.25%	3	15.0%	0.190

CSF: Cerebrospinal fluid. VDRL: Venereal disease research laboratory. ^a^According to Brazilians Ministry of Health adequate treatment for syphilis in pregnancy is 7.2 million international units of penicillin G benzathine

^b^A child who presented any of the following clinical manifestations related to early CS was considered symptomatic at birth: prematurity, low birth weight, hepatomegaly with or without splenomegaly, skin lesions, jaundice with phototherapy or serosanguinolent rhinitis

^c^This variable included both reactive VDRL in the cerebrospinal fluid and alterations in cytology and/or protein analysis suggestive of neurosyphilis

Review of newborn medical records from the 68 children identified two children (3.0%), both from the penicillin treatment group, who were considered to have an altered ophthalmological neonatal triage test, while still at maternity. These infants were referred to ophthalmology for further evaluation. One had retinopathy of prematurity (ROP) confirmed and treated, the other had alterations in visual axis ruled out after the ophthalmologist’s assessment. No other ophthalmological findings were registered during the routine ophthalmological assessment of children with CS.

At the time of the re-evaluation, children had a median age of 8 years (7–8). In this new ophthalmological assessment 8 years later, the majority of children had a completely normal ophthalmological examination (46/67.6%). Similar percentages in the penicillin (5/10.5%) group and the ceftriaxone (2/10%) group had minor refractive errors on examination, but another three (6.3%) patients in the penicillin group were considered highly myopic. Regarding findings that are more associated with CS, one child in ceftriaxone group (5.0%) had optic atrophy in the right eye and one in the penicillin group (2.1%) had glaucomatous optic disc changes and referred for further evaluation (Table 2), no interstitial keratitis was found. Additionally, strabismus was also seen in one child (2.1%) in the penicillin group, which had already been investigated and attributed to an arachnoid cyst in the midbrain and third ventricle region.

**Table 2:** Ophthalmological findings of children with CS after treatment administered in the maternity ward

	Treatment			
	Penicillin (*n* = 48)		Ceftriaxone (*n* = 20)	
	Count	%	Count	%
Normal	31	65,1%	15	75%
Minor refractive error	5	10,5%	2	10%
Highly myopic^a^	3	6,3%	0	0%
Optic atrophy	0	0%	1	5%
Glaucomatous optic disc^b^	1	2,1%	0	0%
Strabismus	1	2,1%	0	0%
ROP Scars	1	2,1%	0	0%
Not evaluated^c^	6	12,6%	2	10%

^a^Myopia equal to or greater than − 6.00 diopters. ^b^Increased cupping and asymmetry of the cup-to-disc ratio. ^c^Patients who were uncooperative or did not attend the ophthalmologic evaluation, are not included in the primary outcome statistics

A new VDRL was performed on all participants. Two children treated with penicillin (5.8%) and one with ceftriaxone (5%) had a reactive VDRL test. The VDRL titration of all of this children (*n* = 3) was 1:1 and each were referred for treatment with penicillin and evaluation of the CSF. HIV tests were also carried out on all the participating children, but none were positive.

There was no significant association between the child’s treatment and the prevalence of ophthalmologic findings (OR: 0.353; CI: 0.039–3.167; *p* = 0.663). There was also no association between the medication and a current reactive VDRL (*p* = 1.000).

## Discussion

In this study involving 68 children diagnosed with CS and treated with either penicillin or ceftriaxone, no significant difference was observed in the prevalence of ophthalmologic manifestations eight years post-treatment.

Neurological involvement is common among symptomatic neonates with CS, with more than 60% presenting signs compatible with neurosyphilis, including ophthalmologic abnormalities. Historically, ophthalmic complications—such as interstitial keratitis and congenital cataracts—have been reported in 5–40% of inadequately treated CS cases [10].

Although ocular syphilis can manifest at any stage of life [3, 4] classical studies suggest a peak incidence during late childhood, between ages 6 and 15 years [4]. In our cohort, only two cases among the 68 children evaluated presented ophthalmologic abnormalities potentially attributable to CS, reinforcing the hypothesis that such complications are relatively infrequent when adequate treatment is provided. No significant difference in ophthalmological findings was detected between treatment groups, suggesting comparable efficacy of ceftriaxone and penicillin in preventing long-term ocular sequelae.

Recent global shortages of penicillin and the complexity of desensitization procedures in allergic patients have stimulated interest in alternative treatments for syphilis [6, 13]. In vitro susceptibility studies have demonstrated low minimum inhibitory concentrations (MICs) for Treponema pallidum with ceftriaxone and other cephalosporins, indicating promising antibacterial activity [5]. Furthermore, ceftriaxone exhibits advantageous pharmacokinetics, such as high liposolubility and enhanced penetration across the blood-brain and blood-ocular barriers, which may contribute to its efficacy in treating neurosyphilis and potentially ocular syphilis [5, 13].

Some observational studies have explored the efficacy of ceftriaxone compared to penicillin (Table 3) in the context of ocular syphilis in adults, with or without HIV co-infection, unanimously suggesting similar results between the drugs. However, there are no studies in CS or in neonates and in this population concerns persist regarding the risk of kernicterus associated with ceftriaxone use, despite emerging evidence suggesting a safety profile comparable to other antibiotics traditionally considered safe for neonates [18].

**Table 3:** Summary of findings of studies evaluating ceftriaxone in comparison to penicillin for ocular syphilis treatment

Author, year	Country	Study design	*N*	Coinfected with HIV %	Mean/median age in years	Clinical response	Odds ratio (CI)
Alhawsawi, 2025 [11]	Saudi Arabia	Retrospective cohort	19	26.3%	40.6	C: 2/2 P:10/10	NA
Bettuzzi, 2021 [12]	France	Retrospective cohort	365	27.9%	44.4	C: 41/42* P: 125/166*	1·22 (1.12–1.33)**
Gu, 2024 [13]	China	Retrospective cohort	205	3.4%	56	C: 13/34* P: 82/171*	0,67 (0.31-1,42)
Gutierrez, 2017 [14]	France	Case series	12	16.6%	57.4	C: 6/6 P: 6/6	NA
Philippe, 2021 [15]	France	Case series	18	22%	48	C: 9/9 P: 6/6 D: 3/3	NA
Puech, 2010 [16]	France	Case series	8	62.5%	52.2	C: 7/7 P: 1/1	NA
Wagner, 2021 [17]	Germany	Retrospective cohort	23	22%	45	C: 4/7* P: 3/8*	NA

N: number of participants in the study. CI: 95% confidence interval. NA: not available. C: ceftriaxone. P: penicillin. D: oral doxycycline. *Clinical and serological response. ** Results for the 1-month follow up, on 6-month follow-up there was no difference between treatments, except in length of hospitalization which was shorter in ceftriaxone

Several limitations must be acknowledged. First, recruitment was restricted to two districts of Fortaleza, which introduced a selection bias, and despite active outreach efforts, not all eligible children could be assessed, which can introduce a non-response bias. The relatively low incidence of ophthalmic complications in CS naturally constrains the statistical power of small studies. However, it is generally expected that inadequately treated cases would manifest higher rates of such complications. Therefore, while our findings suggest similar ophthalmic outcomes between ceftriaxone- and penicillin-treated groups, we cannot definitively rule out a type II error due to the limited sample size.

Despite these limitations, this study contributes valuable data to the growing body of evidence supporting the use of ceftriaxone for CS treatment. It also reinforces the need for long-term, multidisciplinary follow-up of children with CS to detect and manage potential complications, including ocular sequelae.

## Conclusion

In this limited cohort, no significant differences were observed in the incidence of ophthalmologic manifestations between children treated with penicillin and those treated with ceftriaxone for CS after an 8-year follow-up period. Randomized controlled trials comparing standard penicillin-based regimens with ceftriaxone in the neonatal population are warranted to further assess safety, therapeutic efficacy, and long-term outcomes in the prevention of CS complications.

## Data Availability

The datasets used and analysed during the current study are available from the corresponding author on reasonable request.
